# Impact of heat treatment on *Dirofilaria immitis* antigen detection in shelter dogs

**DOI:** 10.1186/s13071-017-2443-7

**Published:** 2017-11-09

**Authors:** Brian A. DiGangi, Carly Dworkin, Jason W. Stull, Jeanette O’Quin, Morgan Elser, Antoinette E. Marsh, Lesli Groshong, Wendy Wolfson, Brandy Duhon, Katie Broaddus, Elise N. Gingrich, Emily Swiniarski, Elizabeth A. Berliner

**Affiliations:** 1 Department of Small Animal Clinical Sciences, University of Florida, College of Veterinary Medicine, PO Box 142275, Gainesville, FL 32614 USA; 2 Department of Veterinary Preventive Medicine, The Ohio State University, Columbus, OH USA; 3 Humane Society of Boulder Valley, Boulder, CO USA; 4 Department of Veterinary Clinical Sciences, Louisiana State University, School of Veterinary Medicine, Baton Rouge, LA USA; 5 Austin Humane Society, Austin, TX USA; 6 Larimer Humane Society, Fort Collins, CO USA; 7 Pet Orphans of Southern California, Van Nuys, CA USA; 8 Department of Population Medicine and Diagnostic Sciences, Cornell University, Ithaca, NY USA; 9 Present address: Tree House Humane Society, Chicago, IL USA; 10 Present address: ASPCA, PO Box 142275, Gainesville, FL 32614 USA

**Keywords:** Canine, Heartworm, *Dirofilaria immitis*, Antigen test, Diagnosis, Immune complex, Heat treatment

## Abstract

**Background:**

The diagnosis and management of canine heartworm disease is a growing concern for shelter veterinarians. Although the accuracy of commercial antigen test kits has been widely studied, recent reports have renewed interest in antigen blocking as a causative factor for false “no antigen detected” results. The objectives of this study were to determine the prevalence of false “no antigen detected” results in adult dogs entering shelters in northern, southern, and western regions of the country and to identify historical and clinical risk factors for such results.

**Methods:**

Serum samples were evaluated for *Dirofilaria immitis* antigen using a commercially available point-of-care ELISA; samples in which no antigen was detected underwent a heat treatment protocol and repeat antigen testing. Whole blood samples underwent Knott testing to identify the presence of microfilariae. Historical and clinical findings were analyzed using exact logistic regression.

**Results:**

A total of 616 samples were analyzed. Overall prevalence of positive antigen test results (prior to heat treatment) was 7.3% and frequency of false “no antigen detected” results due to antigen blocking (ie, samples with no antigen detected prior to heat treatment and positive after heat treatment) was 5.2%. Among dogs that had no detectable antigen on the initial tests, dogs that had microfilariae detected via modified Knott testing (OR = 32.30, *p*-value = 0.013) and dogs that previously received a heartworm preventive (OR = 3.81, p-value = 0.016) had greater odds of antigen blocking than dogs without these factors. Among dogs that were heartworm positive, those without microfilariae detected had greater odds of antigen blocking than dogs with this factor (OR = 11.84, p-value = 0.0005). Geographic region of origin was significantly associated with occurrence of antigen blocking (*p* = 0.0036); however, blocking occurred in all regions sizably contributing to heartworm diagnoses. Of the 74 dogs found to be infected with heartworms in this study, 39.2% (29) had no detectable antigen prior to heat treatment.

**Conclusions:**

Heat treatment of serum samples should be considered to improve diagnostic test accuracy, particularly in dogs that reportedly received a heartworm preventive prior to antigen testing regardless of region of origin.

**Electronic supplementary material:**

The online version of this article (10.1186/s13071-017-2443-7) contains supplementary material, which is available to authorized users.

## Background

The diagnosis and management of canine heartworm disease is a growing concern for shelter veterinarians nationwide [1–4]. A recent survey of shelter veterinarians indicated that the majority of shelter practitioners rely exclusively on point-of-care antigen testing for the diagnosis of *Dirofilaria immitis* in shelter dogs [5]. The results of these tests often determine the management of the dog throughout its stay in the shelter system, including its likelihood of a live release. Given the emphasis on such diagnostic testing for the determination of heartworm-related medical and management decisions in the shelter environment, ensuring accuracy of the testing methodology is of the utmost importance.

Although the accuracy of commercially available antigen test kits has been widely studied, recent reports have renewed interest in causative factors for false “no antigen detected” (NAD) test results [6–8]. One explanation for such results is the phenomenon of antigen blocking due to immune complex formation within the host. Although unknown whether such immune complexes are specific to *D. immitis*, their presence can hinder detection of the parasite in as many as 7% of samples [8]. Destruction of immune complexes through heat treatment of samples prior to testing reduces this occurrence and improves test accuracy. In addition, serum with NAD test results in highly microfilaremic dogs has also been shown to test positive after heat treatment [6].

Given the geographic variation in prevalence of heartworm infection as well as in other disease characteristics (eg, anthelmintic resistance, vector species), it is anticipated that there may be geographic differences in the occurrence of antigen blocking [8]. Determining the prevalence of antigen blocking in populations of shelter dogs and identifying risk factors for false NAD test results are important to elevate the care of shelter dogs and better prepare sheltering organizations and future adopters to meet medical needs.

The objectives of the current study were to 1) determine the prevalence of false NAD antigen tests due to antigen blocking in shelter dogs in three different regions of the United States, 2) identify clinical and historical risk factors for antigen blocking, and 3) to assess the feasibility of a simple, in-shelter heat treatment protocol to disrupt immune complex formation and subsequent false NAD test results. We hypothesized that overall prevalence of positive antigen test results will vary between geographic regions (ie, highest in southern regions, followed by western and northern) and that the frequency of antigen blocking would mirror regional prevalence. In addition, it was expected that microfilaremia would result in greater immune complex formation and therefore increased risk of false NAD antigen test results. Finally, a simple, in-shelter heat treatment protocol would be an effective method of disrupting immune complex formation and improving accuracy of test results.

## Methods

Animal shelters in Florida, Louisiana, and Texas (South); Ohio and New York (North); and Colorado and California (West) were recruited to participate in this study in order to represent three geographic regions with similar reported regional prevalence of canine heartworm infection [9]. Individual shelters were selected based on their willingness to collect and test samples according to the study protocol and the ability to collect a sufficient number of samples during the data collection period (June–November 2015).

Adult dogs six months of age or older (based on known age or estimated by dentition) were eligible for inclusion. Historical data were collected for each dog including the state of origin, intake type (ie, stray, relinquished, seizure, adoption return), whether the dog was transferred from another geographic region, and history of heartworm prophylaxis administered by a previous owner or shelter of origin. Dogs transferred from regions other than the study site were analyzed using their region of origin. Clinical data for each dog were also collected at the time of intake including age, sex and neuter status, predominant breed, body condition score, signs of infectious disease, and signs of noninfectious disease. A uniform data collection log was completed for each animal. Generally, this information was collected by a member of the medical staff within 24 h of admission.

A minimum of three milliliters of whole blood was collected from each dog. One milliliter was placed in EDTA and refrigerated at 1.6–4.4 °C (35–40 °F) until shipment to the diagnostic laboratory where samples underwent Knott testing with morphologic examination and estimation of microfilarial count within 7 days of collection [10]. The remaining blood sample was placed in a sterile collection tube with no additive and allowed to clot. Once separated, the serum was removed and placed in a new sterile tube in preparation for antigen testing. Unless tested immediately, serum samples were refrigerated at 1.6–4.4 °C (35–40 °F) and tested within 7 days of collection.

Antigen testing was conducted with a commercially available ELISA antigen test kit (DiroCHEK® Heartworm Antigen Test Kit, Zoetis) according to the manufacturer’s instructions. Sera from NAD samples were diluted with an equal volume of 0.9% saline to minimize coagulation (personal communication, Susan E. Little). A 500 mL beaker was filled with 250 mL of water, placed in a microwave, and heated to the point of boiling (approximately 2 min in a 1000 W unit). The heated water was removed from the microwave and up to five diluted serum samples were immersed in the hot water bath for 10 min. After heating, each sample underwent repeat antigen testing as previously described.

Prior to treating study samples, two serum samples known to exhibit antigen blocking on previous testing (personal communication, Susan E. Little) underwent the heat treatment protocol in order to confirm its ability to disrupt immune complex formation. Both samples tested NAD prior to and positive after undergoing the heat treatment protocol used in this study.

Data were analyzed using Intercooled Stata version 12.1 (StataCorp, College Station, Texas). Prior to data analysis, dog breeds were categorized based on American Kennel Club classification groups and signs of infectious or noninfectious diseases were categorized according to primary organ system affected. Prevalence of positive antigen test results and frequency of false NAD results due to antigen blocking were calculated. Regional prevalence estimates were determined along with accompanying binomial exact 95% confidence intervals (CI).

Univariable exact logistic regression analyses were performed to identify variables that were significant predictors for antigen blocking. In order to fully investigate antigen blocking, separate analyses were performed comparing dog samples exhibiting antigen blocking (outcome) to two different comparison groups – heartworm-positive samples not exhibiting antigen blocking and true NAD samples. The continuous variables age and body condition score were converted to dichotomous or ordinal variables (<2 years of age, ≥ 2 years of age; 1–3, 4–6, 7–9 years of age); cut-points were based on distribution of the data and biological relevance. Variables significantly (*p* < 0.05) associated with antigen blocking in univariable analysis were eligible for a multivariable exact logistic regression model. Spearman rank correlation coefficient was computed for all pairs of predictor variables to identify high collinearity/correlation (absolute value of rho ≥0.8). If two variables were highly correlated, the variable with the greatest biological plausibility was retained. A manual step-wise forward building procedure was used to create a multivariable model for antigen blocking. As variables were added, likelihood ratio tests were used to assess the significance of each model. Variables were retained if they were significant (p < 0.05) predictors for antigen blocking in the final model. Given the study hypothesis, dog region of origin was forced into the final model (if not already present) to assess significance and confounding (ie, inclusion of the variable changed the coefficient of a significant predictor variable in the model by 25% or more). Odds ratios (OR) and 95% CI for the ORs were calculated for all variables.

## Results

Samples from 616 dogs were eligible for inclusion (see Additional file 1). A total of 45 samples (7.3%, CI = 5.4–9.7) tested positive for *D. immitis* antigen prior to heat treatment. Most positive samples originated from the South (38 [15.9%], CI = 11.5–21.2), followed by those from the West [4 (2.2%), CI = 0.6–5.4] and the North [3 (1.6%), CI = 0.3–4.5].

A total of 558 samples underwent the heat treatment protocol. Twenty-nine samples (5.2%, CI = 3.5–7.4) that initially tested NAD tested positive after heat treatment (Table 1. Results after heat treatment were unavailable for 13 (2.3%) of these samples; five solidified after heat treatment, four had insufficient serum for retesting, three displayed an invalid negative control (with insufficient volume for retesting), and one sample exhibited hemolysis that hindered test interpretation. Final prevalence of antigen-positive test results including heat-treated positive samples was 12.3% (74/603, CI = 9.8–15.2), representing detection of an additional 29 positive samples or a 64.4% increase in detection as compared with detection without heat treatment. Overall regional prevalence was 22.7% (CI = 17.4–28.7) in the South, 7.1% (CI = 3.8–11.8) in the West, and 4.7% (CI = 2.2–8.8) in the North.

**Table 1:** Frequency of false “no antigen detected” test results after heat treatment of serum samples from 558 dogs

Region	N	Number false NAD (%)	95% CI^a^
All	558	29 (5.2)	3.5, 7.4
North	188	6 (3.2)	1.2, 6.8
South	191	14 (7.3)	4.0, 12.0
West	179	9 (5.0)	2.3, 9.3

^a^Binomial exact 95% confidence interval

After modified Knott testing, *D. immitis* microfilariae were detected in 26 (4.2%) of the 616 samples. Two of these microfilariae positive samples tested NAD for *D. immitis* antigen prior to but positive following heat treatment. *Acanthocheilonema reconditum* was detected in six samples (representing samples from the South [3], North [2], and West [1]), all of which tested NAD for *D. immitis*.

Potential risk factors for false NAD test results (antigen blocking) were evaluated (see Additional file 2 **)**. Of dogs that were initially NAD, those that had a reported history of heartworm preventive administration (OR = 4.15, *p* = 0.006) and those with circulating *D. immitis* microfilariae (OR = 45.37, *p* = 0.005) exhibited significantly greater odds of antigen blocking than those without such findings. Both microfilarial status and history of heartworm preventive administration remained in the final multivariable model; dogs that had a reported history of heartworm preventive administration (OR = 32.30, 95% CI = 2.20–infinity, *p* = 0.013) and those with circulating *D. immitis* microfilariae (OR = 3.81, 95% CI = 1.29–10.05, *p* = 0.016) exhibited significantly greater odds of antigen blocking than those without such findings. Region of origin was not a confounder or significant predictor in the final model, and thus was removed.

Potential risk factors for antigen blocking in dogs that were heartworm positive were also evaluated (see Additional file 3). Dogs’ region of origin was significantly associated with antigen blocking (*p* = 0.0036). Dogs from the west (69.2% of positive dogs exhibited antigen blocking) had a higher odds of antigen blocking than dogs from the south (26.9%, OR = 5.91, p = 0.01); dogs from the north (66.7%) did not significantly differ from the other regions. Compared with dogs that were initially positive, those without circulating microfilariae (OR = 14.89, 95% CI = 3.12–144.26, *p* < 0.0001) exhibited significantly greater odds of antigen blocking than those with circulating microfilariae. Both region of origin and microfilarial status remained significant in the final multivariable model with similar ORs to univariable models; dogs without circulating *D. immitis* microfilariae (OR = 11.84, 95% CI = 2.42–115.36, *p* = 0.0005) and region of origin [p < 0.0001; no region-to-region comparisons significant (west to south: OR = 4.53, 95% CI = 0.91–28.39, *p* = 0.070)] were significant predictors for antigen blocking.

## Discussion

False NAD test results due to antigen blocking were demonstrated in samples from each region of the United States. Samples from dogs that had circulating *D. immitis* microfilariae and those that were reported to have previously received a heartworm preventive were more likely to exhibit antigen blocking than true NAD samples from dogs without these characteristics. Samples from infected dogs originating from northern and western regions of the country were more likely to exhibit antigen blocking than those from the South. Infected dogs without circulating *D. immitis* microfilariae were more likely to exhibit antigen blocking than those with circulating microfilariae. The overall prevalence of antigen blocking reported here (5.2%, by region 3.2%–7.3%) is lower than that reported in a population of Romanian shelter dogs (18.6%) [11] but similar to that found in another population of shelter dogs from the southeastern United States (7.1%) [8].

To our knowledge this is the first report to evaluate clinical and historical risk factors for antigen blocking in canine serum samples. A previous study in dogs actively being managed with macrocyclic lactones and doxycycline for chronic inflammatory diseases including dirofilariasis found that over 50% of samples exhibited antigen blocking, including one of nine dogs that had circulating microfilaria [12]. In the Romanian study, none of the dogs had received macrocyclic lactones; however, circulating *D. immitis* microfilariae were identified in three dogs that exhibited antigen blocking [11]. Conversion of test results from NAD to positive was also found in four dogs that had circulating *D. repens* microfilariae. The strong association between historical administration of heartworm preventives identified in this report lends further support to the impact of this factor on immune complex formation and subsequent false NAD test results. The findings in the current report regarding microfilarial status were more complex. While more likely to be found in samples exhibiting antigen blocking as compared with those that tested NAD, circulating *D. immitis* microfilariae were less likely to be found in samples from all infected dogs. Because the true status of a dog is not likely to be known in an initial clinical testing scenario, these conflicts highlight the importance of utilizing both antigen and microfilarial testing in the diagnosis of heartworm infection. Samples in which both antigen and microfilariae are unable to be detected in light of clinical and historical findings suggestive of heartworm infection may be candidates for heat treatment and further antigen testing.

This is also the first report to quantify antigen blocking in populations of dogs from geographic regions with differing prevalence of heartworm infection. Although frequency of antigen blocking mirrored prevalence of heartworm infection, it was not found to be significantly different in dogs from different geographic regions when compared with dogs that tested NAD both before and after heat treatment. Given that other factors, such as anthelmintic resistance, have been linked to specific geographic differences in canine heartworm disease [13], this finding suggests that an individual dog’s immunologic response to infection (ie, immune complex formation) is independent from the specific heartworm isolate. When compared with dogs that tested positive prior to heat treatment, however, dogs from origins in the northern and western regions of the country were more likely to exhibit antigen blocking than those from the south. The low frequency of positive test results from dogs in northern and western regions limit interpretation of these findings. Additional evaluation of infected dogs from these regions, including identification of concurrent inflammatory diseases as well as analysis of the immune response generated by regional heartworm isolates, may further elucidate this relationship.

The in-shelter heat treatment protocol was successfully conducted at each study location. The materials used to perform heat treatment were readily available and inexpensive (<$180 was spent on consumable supplies specific to the heat treatment protocol for all 616 samples). Diagnostic testing was time consuming, however, particularly given that the study protocol required testing of most samples twice. Outside of the research protocol, heating all samples prior to initial testing and, for those shelters with fewer samples, storing and testing a large group of samples at one time may improve feasibility of in-shelter heat treatment.

There are several limitations to the findings reported in this study. Although the testing protocol was standardized, antigen tests were conducted onsite at each location by a different investigator. Consistency of the heat treatment protocol (ie, temperature and duration of treatment) could not be assured. Previous studies ensured consistency of heat treatment through the use of a dry heat block maintained at a precise temperature for (103–104 °C) for 10 min [7, 8]. It is plausible that some samples may not have reached a high enough temperature for a long enough period of time to disrupt immune complexes; however, all but one study site had at least one sample that converted from NAD to positive after treatment. The site that did not detect any positive samples even after heat treatment was located in the northeastern part of the country with an expected heartworm prevalence of less than 1%. Conversely, the five samples that solidified after heat treatment were presumed to have overheated, making them unable to be tested after immune complex disruption. Second, antigen test results relied on subjective evaluation of colorimetric changes. To maintain feasibility in the shelter setting, spectrophotometric or optical density recordings were not utilized. False-positive results are possible, particularly if test results were not read promptly after completing the test procedure. Third, only instances of heartworm preventive administration that were verifiable were included. It is possible that records from dogs transferred from other facilities or histories provided by owners relinquishing their pets were inaccurate or missing regarding previous preventive administration. Fourth, given the small sample sizes encountered, exact methods were used for analyses. Such methods often have reduced power to detect true differences in groups and may lead to unstable point estimates (as observed with wide confidence intervals). Finally, given that this study utilized serum samples from dogs undergoing routine screening tests in preparation for adoption programs, accuracy of test results could not be definitively confirmed. Necropsies were not performed to confirm test results nor were known negative samples (ie, those from specific-pathogen free dogs or those <4 months of old) included in the analysis.

The antigen test kit used in this study was chosen due to its well-established high level of sensitivity [14]. False NAD test results obtained with other testing methodologies could be attributed to lower test sensitivity rather than antigen blocking alone. For this reason, the current results should not be extrapolated to those obtained with different antigen test kits, although there is no reason to expect that antigen blocking would not interfere with other ELISA-based tests. Reports indicate that 65% to 95% of shelters rely on a single antigen test to determine heartworm infection status in their dogs [5, 15], so further evaluation on the impact of other antigen testing methodologies is warranted prior to regular clinical use of a heat treatment protocol.

## Conclusions

Specific clinical and historical risk factors for antigen blocking were identified: a history of heartworm preventive administration and the presence of circulating *D. immitis* microfilariae. This latter finding supports the recommendation for tandem testing for both antigen and microfilariae [16]. The simple heat treatment protocol evaluated was feasible for use in a shelter setting, though more controlled heating might allow for additional “testable” samples. Heat treatment of serum samples increased heartworm detection by over 60% in this study and should be considered to improve accuracy of diagnostic test results in select circumstances.

## Additional files

Additional file 1: Demographic information of 616 shelter dogs tested for *Dirofilaria immitis. (DOCX 14 kb)*

Additional file 2: Univariable exact logistic regression for antigen blocking in dogs that initially had no detectable antigen with a point-of-care ELISA (*n* = 558). (DOCX 13 kb)

Additional file 3: Univariable exact logistic regression for antigen blocking in dogs that were heartworm positive with a point-of-care ELISA with and without heat treatment (*n* = 74). (DOCX 20 kb)

## Abbreviations

BCS: Body condition score
CI: Confidence interval
EDTA: Ethylenediaminetetraacetic acid
ELISA: Enzyme-linked immunosorbent assay
NAD: No antigen detected
OR: Odds ratio
SD: Standard deviation
