# Supplementary material for: Impact of heat treatment on Dirofilaria immitis antigen detection in shelter dogs
Source: Parasit Vectors. 2017 Nov 9;10(Suppl 2):483. doi: 10.1186/s13071-017-2443-7 (PMC5688474; doi:10.1186/s13071-017-2443-7)
Supplement: Supplementary file 1 — Demographic information of 616 shelter dogs tested for Dirofilaria immitis. (DOCX 14 kb) [file 13071_2017_2443_MOESM1_ESM.docx]

**Additional file 1 – Demographic information of 616 shelter dogs tested for *Dirofilaria immitis***

| Variable | | | N | Count (%)  [unless otherwise noted] |
| --- | --- | --- | --- | --- |
| Age (years) | | | 612 |  |
|  | Mean (SD) | |  | 3.0 (2.6) |
|  | Median | |  | 2.0 |
|  | Range | |  | 0.5-15.0 |
| Sex | | | 615 |  |
|  | Female | |  | 288 (46.8) |
|  | Male | |  | 327 (53.2) |
| Neutered | | | 612 |  |
|  | No | |  | 362 (59.2) |
|  | Yes | |  | 250 (40.8) |
| Breed group | | | 608 |  |
|  | Herding | |  | 48 (7.9) |
|  | Hound | |  | 56 (9.2) |
|  | Non-sporting | |  | 31 (5.1) |
|  | Other | |  | 165 (27.1) |
|  | Sporting | |  | 108 (17.8) |
|  | Terrier | |  | 49 (8.1) |
|  | Toy | |  | 97 (16.0) |
|  | Working | |  | 54 (8.9) |
| Body condition score | | | 457 |  |
|  | 1-3 | |  | 44 (9.6) |
|  | 4-6 | |  | 367 (80.3) |
|  | 7-9 | |  | 46 (10.1) |
| Infectious disease | | | 614 |  |
|  | No | |  | 569 (92.7) |
|  | Yes^a^ | |  | 45 (7.3) |
|  |  | Dermatologic |  | 1 |
|  |  | Gastroenteric |  | 7 |
|  |  | Ophthalmologic |  | 14 |
|  |  | Respiratory |  | 31 |
|  |  | Other* |  | 1 |
| Noninfectious disease | | | 614 |  |
|  | No | |  | 390 (63.5) |
|  | Yes^a^ | |  | 224 (36.5) |
|  |  | Dermatologic |  | 108 |
|  |  | Gastroenteric |  | 6 |
|  |  | Ophthalmologic |  | 21 |
|  |  | Other** |  | 116 |
| Ectoparasites visualized^b^ | | | 613 |  |
|  | No | |  | 550 (89.7) |
|  | Yes | |  | 63 (10.3) |
| Intake type | | | 615 |  |
|  | Adoption Return | |  | 18 (2.9) |
|  | Owner Surrender | |  | 208 (33.8) |
|  | Seizure | |  | 19 (3.1) |
|  | Stray | |  | 313 (50.9) |
|  | Other | |  | 49 (8.0) |
|  | Unknown | |  | 8 (1.3) |
| Arrived via transport program | | | 615 |  |
|  | No | |  | 568 (92.4) |
|  | Yes | |  | 47 (7.6) |
| Region^c^ | | | 616 |  |
|  | North | |  | 191 (31.0) |
|  | South | |  | 239 (38.8) |
|  | West | |  | 186 (30.2) |
| History of previous heartworm preventive administration | | | 613 |  |
|  | No | |  | 553 (90.2) |
|  | Yes | |  | 60 (9.8) |
| Preventive administered by shelter prior to sampling | | | 615 |  |
|  | No | |  | 372 (60.5) |
|  | Yes | |  | 243 (39.5) |
| Microfilariae | | | 616 |  |
|  | Absent | |  | 590 (95.8) |
|  | Present^d^ | |  | 26 (4.2) |
| Microfilariae (*D. immitis,* count)^e^ | | | 26 |  |
|  | Median | |  | 16 |
|  | Range | |  | 1-202 |

^a^More than one system could be reported

^b^Inconsistently reported by shelters

^c^Categorized by state of origin

^d^DiroCHEK^®^ negative (n=2; both positive with heat treatment); DiroCHEK^®^ positive (n=24)

^e^Count calculated as median of three estimates each based on five 10x fields

*One dog had fever and conjunctivitis

**Dogs had one or more of the following conditions: abnormal biochemistry analysis, brachycephalic syndrome, dental disease, endocrinopathy, fever, heart murmur, infection, lymphadenopathy, muscle wasting, neoplasia, neurologic disease, orthopedic disease, severe matting, superficial wound or injury
