# Supplementary material for: Impact of heat treatment on Dirofilaria immitis antigen detection in shelter dogs
Source: Parasit Vectors. 2017 Nov 9;10(Suppl 2):483. doi: 10.1186/s13071-017-2443-7 (PMC5688474; doi:10.1186/s13071-017-2443-7)
Supplement: Supplementary file 2 — Univariable exact logistic regression for antigen blocking in dogs that initially had no detectable antigen with a point-of-care ELISA (n = 558). (DOCX 13 kb) [file 13071_2017_2443_MOESM2_ESM.docx]

**Additional file 2 – Univariable exact logistic regression for antigen blocking in dogs that initially had no detectable antigen with a point-of-care ELISA (n=558)**

| Variable | | N | No. positive/no. tested (%) | OR (95% CI) | p-value |
| --- | --- | --- | --- | --- | --- |
| Age | | 554 |  |  |  |
|  | < 2 yr. |  | 12/210 (5.7) | Referent |  |
|  | ≥ 2 yr. |  | 17/344 (4.9) | 0.86 (0.38, 2.01) | 0.83 |
| Sex | | 557 |  |  |  |
|  | Female |  | 12/256 (4.7) | Referent |  |
|  | Male |  | 17/301 (5.7) | 1.22 (0.54, 2.85) | 0.76 |
| Neutered | | 554 |  |  |  |
|  | No |  | 14/324 (4.3) | Referent |  |
|  | Yes |  | 15/230 (6.5) | 1.54 (0.68, 3.53) | 0.34 |
| Body condition score | | 403 |  |  | 0.14 |
|  | 1–3 |  | 5/38 (13.2) | Referent |  |
|  | 4–6 |  | 17/323 (5.3) | 0.37 (0.12, 1.36) | 0.14 |
|  | 7-9 |  | 2/42 (4.8) | 0.33 (0.030, 2.21) | 0.35 |
| Infectious disease | | 556 |  |  |  |
|  | No |  | 27/513 (5.3) | Referent |  |
|  | Yes |  | 2/43 (4.7) | 0.88 (0.10, 3.72) | 1.00 |
| Noninfectious disease | | 556 |  |  |  |
|  | No |  | 21/343 (6.1) | Referent |  |
|  | Yes |  | 8/213 (3.8) | 0.60 (0.23, 1.44) | 0.31 |
| Ectoparasites visualized | | 555 |  |  |  |
|  | No |  | 25/493 (5.1) | Referent |  |
|  | Yes |  | 4/62 (6.5) | 1.3 (0.32, 3.93) | 0.82 |
| Arrived via transport program | | 557 |  |  |  |
|  | No |  | 26/511 (5.1) | Referent |  |
|  | Yes |  | 3/46 (6.5) | 1.3 (0.24, 4.5) | 0.87 |
| Region | | 558 |  |  | 0.19 |
|  | North |  | 6/188 (3.2) | Referent |  |
|  | South |  | 14/191 (7.3) | 2.40 (0.84, 7.78) | 0.11 |
|  | West |  | 9/179 (5.0) | 1.60 (0.50, 5.60) | 0.53 |
| History of previous heartworm preventive administration | | 554 |  |  |  |
|  | No |  | 21/502 (4.2) | Referent |  |
|  | Yes |  | 8/52 (15.4) | 4.15 (1.50, 10.47) | 0.0064 |
| Microfilariae (*D. immitis)*^b^ | | 558 |  |  |  |
|  | Absent |  | 27/556 (4.9) | Referent |  |
|  | Present |  | 2/2 (100) | 45.37^a^ (3.50, Infinity) | 0.0052 |

^a^Median unbiased estimate

^b^Referent group varies between Tables 3 and 4 so that calculated OR estimates are greater than 1 when possible to facilitate interpretation

See Additional file 1 for remainder of key.
