# Supplementary material for: Impact of heat treatment on Dirofilaria immitis antigen detection in shelter dogs
Source: Parasit Vectors. 2017 Nov 9;10(Suppl 2):483. doi: 10.1186/s13071-017-2443-7 (PMC5688474; doi:10.1186/s13071-017-2443-7)
Supplement: Supplementary file 3 — Univariable exact logistic regression for antigen blocking in dogs that were heartworm positive with a point-of-care ELISA with and without heat treatment (n = 74). (DOCX 20 kb) [file 13071_2017_2443_MOESM3_ESM.docx]

**Additional file 3 – Univariable exact logistic regression for antigen blocking in dogs that were heartworm positive with a point-of-care ELISA with and without heat treatment (n=74)**

| Variable | | N | No. Ag-block/no. HW-positive (%) | OR (95% CI) | p-value |
| --- | --- | --- | --- | --- | --- |
| Age | | 74 |  |  |  |
|  | < 2 yr. |  | 12/23 (52.2) | Referent |  |
|  | ≥ 2 yr. |  | 17/51 (33.3) | 0.46 (0.15, 1.41) | 0.20 |
| Sex | | 74 |  |  |  |
|  | Female |  | 12/38 (31.6) | Referent |  |
|  | Male |  | 17/36 (47.2) | 1.92 (0.68, 5.58) | 0.25 |
| Neutered | | 74 |  |  |  |
|  | No |  | 14/46 (30.4) | Referent |  |
|  | Yes |  | 15/28 (53.6) | 2.60 (0.89, 7.80) | 0.08 |
| Body condition score | | 65 |  |  | 0.65 |
|  | 1-3 |  | 5/11 (45.5) | Referent |  |
|  | 4-6 |  | 17/50 (34.0) | 0.62 (0.14, 2.98) | 0.70 |
|  | 7-9 |  | 2/4 (50.0) | 1.19 (0.06, 22.29) | 1.00 |
| Infectious disease | | 74 |  |  |  |
|  | No |  | 27/70 (38.6) | Referent |  |
|  | Yes |  | 2/4 (50.0) | 1.58 (0.11, 23.03) | 1.00 |
| Noninfectious disease | | 74 |  |  |  |
|  | No |  | 21/55 (38.2) | Referent |  |
|  | Yes |  | 8/19 (42.1) | 1.17 (0.35, 3.83) | 0.97 |
| Ectoparasites visualized | | 74 |  |  |  |
|  | No |  | 25/69 (36.2) | Referent |  |
|  | Yes |  | 4/5 (80.0) | 6.86 (0.63, 354.37) | 0.15 |
| Arrived via transport program | | 74 |  |  |  |
|  | No |  | 26/71 (36.6) | Referent |  |
|  | Yes |  | 3/3 (100) | 6.34^a^ (0.66, Infinity) | 0.11 |
| Region^b^ | | 74 |  |  | 0.0036 |
|  | North |  | 6/9 (66.7) | Referent |  |
|  | South |  | 14/52 (26.9) | 0.19 (0.03, 1.03) | 0.056 |
|  | West |  | 9/13 (69.2) | 1.12 (0.12, 9.62) | 1.00 |
| History of previous heartworm preventive administration | | 74 |  |  |  |
|  | No |  | 21/58 (36.2) | Referent |  |
|  | Yes |  | 8/16 (50.0) | 1.75 (0.49, 6.25) | 0.47 |
| Microfilariae (*D. immitis*)^c^ | | 74 |  |  |  |
|  | Absent |  | 27/48 (56.3) | 14.89 (3.12, 144.26) | < 0.0001 |
|  | Present |  | 2/26 (7.7) | Referent |  |

^a^Median unbiased estimates

^b^Comparison for non-referent categories: Dogs from the West region had greater odds of antigen blocking than those from the South (OR= 5.91; 95% CI: 1.38, 30.69; p = 0.01).

^r^Referent group varies between Additional files 2 and 3 so that calculated OR estimates are greater than 1 when possible to facilitate interpretation

See Additional file 1 for remainder of key.
